# Supplementary material for: Interactions of the protein tyrosine phosphatase PTPN3 with viral and cellular partners through its PDZ domain: insights into structural determinants and phosphatase activity
Source: Front Mol Biosci. 2023 May 2;10:1192621. doi: 10.3389/fmolb.2023.1192621 (PMC10185773; doi:10.3389/fmolb.2023.1192621)
Supplement: Supplementary file 1 [file DataSheet2.PDF]

```
>P04843
ILDAL
>P11021
EKDEL
>Q3ZCM7
EEEVA
>Q15084
GKDEL
>P13010
LLDMI
>P22695
FVDEL
>P14625
EKDEL
>P20674
GLDKV
>P62826
EDDDL
>P48047
MREIV
>Q71DI3
RGERA
>O75947
PIENL
>P13639
FLDKL
>P27797
AKDEL
>Q14257
YHDEL
>P14314
DHDEL
```
