# Supplementary material for: Interactions of the protein tyrosine phosphatase PTPN3 with viral and cellular partners through its PDZ domain: insights into structural determinants and phosphatase activity
Source: Front Mol Biosci. 2023 May 2;10:1192621. doi: 10.3389/fmolb.2023.1192621 (PMC10185773; doi:10.3389/fmolb.2023.1192621)
Supplement: Supplementary file 2 [file DataSheet4.PDF]

```
>Q9Y2T3
FSSSV
>P49189
VESAF
>Q9HB71
GDTEF
>Q15046
VGTSV
>O43707
GESDL
>O95336
KHSTL
>P20020
LETSL
>P12814
GESDL
>P23634
LETSV
>P55060
SVTLL
>P27816
QETSI
>P53985
EESPV
>Q96BN8
EETSL
>P35354
RSTEL
>P48960
SESGI
>P52569
KTSEF
>P62829
AGSIA
>Q96TA1
VQTEF
>Q9Y6M5
PESSL
>O15427
PETSV
>P23219
PSTEL
>P28331
EPSIC
>P81605
LDSVL
>O14734
SESKL
>P09543
SCTII
>P11169
TTTNV
>P26599
SKSTI
```

```
>Q13151
GGSSF
>Q5JVF3
LSTVC
>Q8TAA9
SETSV
>Q8TBC3
NETSF
>Q9Y6M7
AETSL
>O00592
EDTHL
>Q02543
PNTFF
```
