# Supplementary material for: Interactions of the protein tyrosine phosphatase PTPN3 with viral and cellular partners through its PDZ domain: insights into structural determinants and phosphatase activity
Source: Front Mol Biosci. 2023 May 2;10:1192621. doi: 10.3389/fmolb.2023.1192621 (PMC10185773; doi:10.3389/fmolb.2023.1192621)
Supplement: Supplementary file 3 [file DataSheet3.PDF]

>P25705
AGFEA
>P08195
FPYAA
>P21796
LEFQA
>P45880
LELEA
>Q9P0L0
GKFIL
>Q9Y277
FELEA
>P35610
CRYVF
>P51571
SHIQA
>P62081
PEFQL
>O00303
KLVNLf
>P46778
YEFMA
>O14975
KTLKL
>O95292
GKIAL
>P16070
MKIGV
>P61619
GALLF
>Q92973
AFYGV
>O75533
LDYIL
>O75964
IGYDV
>O95399
WKYCV
>P00403
PVFTL
>P31930
FWLRF
>P62277
SALVA
>Q59GN2
TKLGL
>Q969Q0
QVIQF
>Q05193
PPFDL
>Q13347
FEFEA
>Q5JTV8
RGICL

>Q7Z2K6
DLFVF
>Q96P70
QTIGI
>Q9NZ01
IPFLL
>Q9P2X0
RGLRF
>Q9UMS4
KFYSL
>O00743
TPYFL
